# Spectral synthesis of temporal response of nonlinearity through tuneable electron and phonon dynamics in a metamaterial

**DOI:** 10.1038/s44310-025-00098-x

**Published:** 2026-01-08

**Authors:** Jingyi Wu, Anton Yu. Bykov, Anastasiia Zaleska, Anatoly V. Zayats

**Affiliations:** Department of Physics and London Centre for Nanotechnology, King’s College London, London, UK

**Keywords:** Metamaterials, Nanophotonics and plasmonics

## Abstract

Manipulating intensity, phase and polarisation of the electromagnetic fields on ultrafast timescales is essential for all-optical switching, optical information processing and development of novel time-variant media. Noble metal based plasmonics has provided numerous platforms for optical switching and control, enabled by strong local field enhancement, artificially engineered dispersion and strong Kerr-type free-electron nonlinearities. However, achieving precise control over switching times and spectral response remains challenging, often limited by hot-electron gas relaxation on picosecond timescales and by the intrinsic band structure of the materials. Here, we experimentally demonstrate a strong and tunable nonlinearity in a metamaterial-on-a-mirror geometry, controlled by the wavelength of excitation, which imprints a specific, non-uniform hot-electron population distribution, driving targeted electron and lattice dynamics. The synergistic exchange of electromagnetic, electronic and mechanical energies enables reflection changes on sub-300 fs timescales in selected spectral ranges, surpassing the limitations imposed by the inherent material response of metamaterial constituents. The observed effect–present in reflection due to leaky guided modes of the metamaterial, but absent in transmission–is highly spectrally selective and sensitive to polarisation of light, opening a pathway to tailoring switching rates through the choice of operating wavelength and nanostructure design. The ability to manipulate temporal, spectral, and mechanical aspects of light-matter interactions underscores new opportunities for nonlinear optical applications where polarisation diversity, spectral selectivity, and ultrafast modulation are important.

## Introduction

Ultrafast optical nonlinearities are highly desirable for numerous applications in laser physics, integrated photonics, active nanophotonics and quantum technologies^1,2^. Dielectric and plasmonic nanostructures and metamaterials are widely used for enhancing nonlinear optical processes. While the nonlinear efficiency is readily controlled by the field enhancement, the temporal response is more difficult to manipulate as it is related to the intrinsic properties of material constituents.

Metasurfaces based on dielectric nanodisk arrays have demonstrated the potential of using multiple nonlinear dynamics to achieve ultrafast all-optical switching in transmission and reflection, which also enables self-modulation of ultrafast pulses^3,4^. Electronic nonlinearities in plasmonic nanostructures are related to rapid heating of the electron gas, which results in the Kerr-type nonlinear response, and provide many opportunities to design the subwavelength-size active photonic devices for enhanced and ultrafast light–matter interaction^5^. Impulsive heating of the electron gas initiates a spectrum of dynamic phenomena and thermal effects in nanostructures that enable ultrafast control over optical waveforms through the nonlinearity of hot-electron gas^5,6^. The modulation rate in this case is limited by characteristic lifetimes of hot-electron population in metal^7,8^; however, nanostructuring was shown to impact the response time by engineering the hot-electron diffusion away from the interfaces^9^.

Mechanical degrees of freedom can also be exploited related to the energy transfer from the hot-electron ensemble to the mechanical motion of nanostructures, resulting in the coherent excitation of acoustic phonons^10,11^. This phenomenon has been observed in both individual nanoparticles^12,13^ and complex nanostructures^14–16^. These excitations can be selectively manipulated through temporal or spectral tailoring of the impinging light^17^ and may interfere with the transient response driven by the Kerr-type nonlinearity of hot electrons. Such interactions provide a distinct and independent mechanism to control the optical properties of nanostructures and are integral to enhancing the efficiency and effectiveness of the metamaterials in nanophotonic devices.

Here, we demonstrate the strong and tuneable nonlinearity in reflection driven by spectrally tunable non-uniform hot-electron population and associated excitation of acoustic vibrations in a plasmonic metamaterial-on-a-mirror geometry. While most of the previous realisations of the enhanced nonlinear response of plasmonic nanorod metamaterials were in transmission in the epsilon-near-zero spectral range, we consider a nonlinear response away from the ENZ wavelength, exploiting reflection resonances linked to leaky waveguided mode excitation of a metamaterial slab. This allows the nonlinear modulation at a virtually arbitrary wavelength and polarisation of the exciting light, demonstrating the nonlinearity enhancement in the hyperbolic nanorod metamaterials for TE-polarised light for the first time, thus eliminating the previous restrictions on nonlinear operation in the ENZ regime with TM-polarised light. We show that by selecting the excitation wavelength, the energy distribution localised in the metamaterial or the mirror to which the metamaterial is connected can be controlled, resulting in the different excitation efficiency of the acoustic phonon modes of the nanorods. The dynamic interaction between photonic, acoustic and electronic modes of the metamaterial allows controlling the response time of the nonlinearity beyond the characteristic timescales of plasmonic hot-electron relaxation alone for arbitrary wavelength and polarisation of pump and probe light.

## Results

**Fig. 1:**
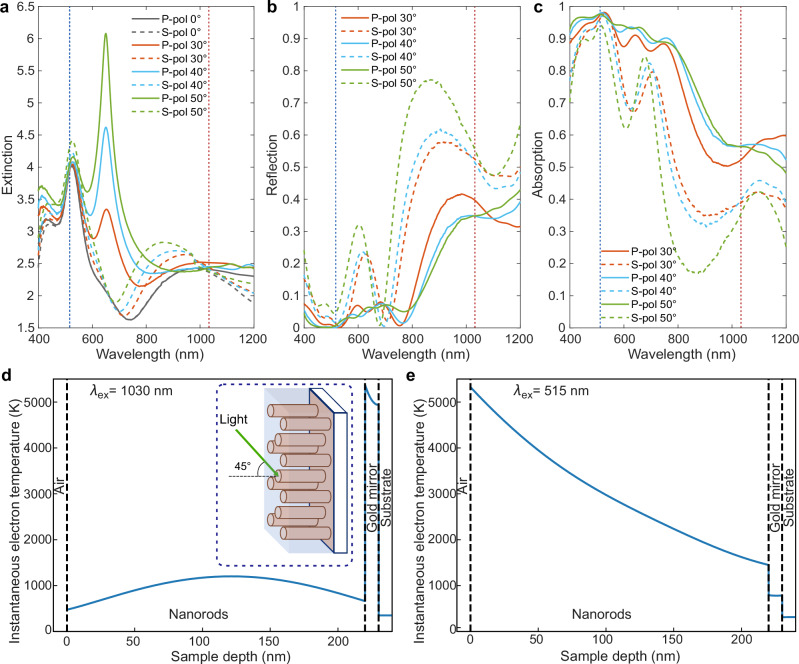
Optical properties of the metamaterial on a mirror system. **a** Extinction, **b** reflection, and **c** absorption of the nanorod metamaterial measured for various polarisations and angles of incidence. The metamaterial parameters are: nanorod radius ≈14 nm, length ≈220 nm and spacing ≈70 nm. Instantaneous hot-electron temperature distributions at different excitation wavelengths: (**d**) 1030 nm and (**e**) 515 nm, normalised to the same absorbed energy density 1 mJ cm^−2^. The vertical lines in (**a**–**c**) indicate the excitation wavelengths used in pump-probe experiments.

We studied hot-electron and phonon dynamics in the metamaterial based on an array of plasmonic (gold) nanorods (length ≈220 nm, diameter ≈28 nm and spacing ≈70 nm) embedded in a dielectric matrix of fused alumina. The metamaterial is grown on a glass substrate and has an 8-nm-thick gold underlayer, achieving a metamaterial on a mirror geometry (Fig. 1d insert; the details of the fabrication process are described in Methods). The extinction peak at the short wavelengths originates from the transverse plasmonic resonance of the nanorods^18^, which can be excited under both *p* (TM)- and *s* (TE)-polarised illuminations (Fig. 1a). The nanorod metamaterial behaves as an optically anisotropic medium and, for the extraordinary wave, exhibits the ENZ condition at a wavelength of approximately 650 nm, where the real part of the effective permittivity *ℜ*{*ϵ*_*z*_} approaches zero. This corresponds to the transition between the elliptic and hyperbolic dispersion regimes of the metamaterial^18,19^. Around this wavelength, a strong extinction peak is observed under oblique incidence with *p*-polarised illumination. The transient nonlinear properties of such hyperbolic metamaterials related to the hot-electron excitation were extensively studied in the past around the ENZ wavelength at which strong enhancement was observed^20–23^. Beyond the ENZ wavelength, the metamaterial exhibits weaker absorption, enabling detailed analysis of the transient processes associated with the leaky guided modes, which can be observed in reflection for both polarisations of light. The reflection spectra (Fig. 1b) exhibit a series of pronounced resonances in the hyperbolic regime (*λ* > *λ*_*ENZ*_), characteristic of leaky waveguided modes of the metamaterial layer^24^, which effectively modulate the absorption spectra (Fig. 1c). The modes correspond to the minima in the reflection spectra (see Fig. S1 for the field distributions of the selected modes at the excitation and probe wavelengths) and above the light line, i.e., accessible by the illumination from the free-space, both TE and TM modes have a low group velocity in the hyperbolic spectral range^24^.

To understand the effect of the mirror to which the metamaterial is connected and which is always present in the experiment due to the fabrication process, we performed numerical simulations of the spectral response of the metamaterial with and without the mirror (Fig. S2). The effect of the mirror is negligible in extinction in the studied spectral range (600–800 nm) but has a profound impact on the reflection spectra (leaky guided modes) and increases absorption.

### Spectrally tuneable hot-electron population

The excitation of optically-thick metamaterials produces the non-uniform temperature distributions within the structure that can be tailored by the excitation wavelength^9,25^. Light absorption governed by the electromagnetic mode structure of the metamaterial placed onto a gold mirror results in the increase of the electron temperature in both the nanorods and the mirror with a strong wavelength-dependent spatial profile (Fig. 1d, e; see Methods for the details of the simulations). Absolute values of the temperature rise are similar for the same powers of the absorbed energy at different wavelengths. However, under the excitation in the NIR spectral range (1030 nm), the gold mirror absorbs most of the optical energy and the electron temperature in the mirror is changed dramatically, while the electron temperature in the metamaterial is almost five times smaller with small variations along the nanorods. The situation is opposite for the excitation in the visible spectral range (515 nm), with the strongest temperature increase observed in the nanorods and five times smaller in the gold mirror. Strong electron temperature gradient is present along the nanorods in this case, with the maximum temperature increase at the illuminated interface.

**Fig. 2:**
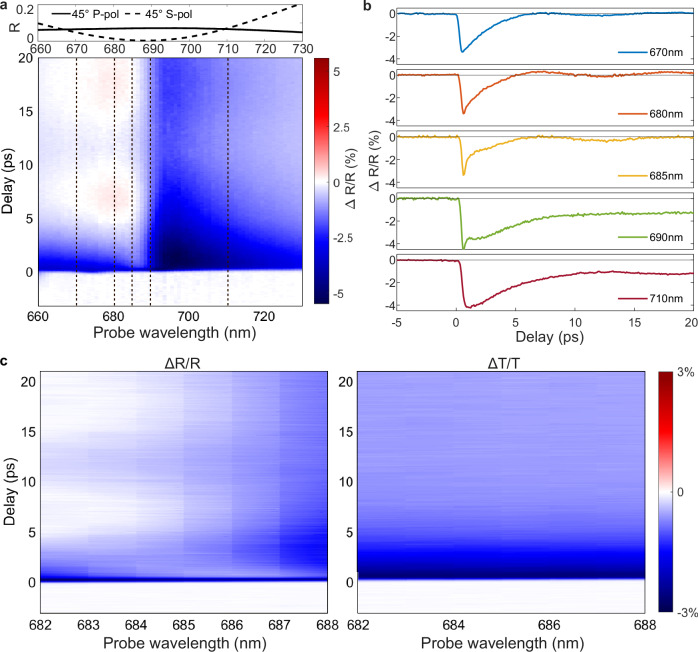
Transient optical spectra of the metamaterial under the 1030 nm excitation. **a** Top: reflection spectra of the metamaterial on a mirror in the ground state measured at 45^∘^ angle of incidence. Bottom: transient reflection spectra measured at the 1030 nm excitation and s-polarised probe light. **b** Time dependent reflectivity at selected wavelengths of 670, 680, 685, 690 and 710 nm (the corresponding cross-sections in (**a**) are indicated with dashed lines). **c** High-resolution transient spectra of (left) reflection and (right) transmission near the resonance, measured simultaneously.

The reflection modulation of nearly 10% under the moderate NIR excitation intensities is achieved due to the increased reflection upon hot-electron generation (Fig. 2a). The strongest change of reflection is observed in the vicinity of the guided mode at a wavelength of 690 nm. (Please note that no significant transient reflection signal was observed in the vicinity of the ENZ wavelength (Fig. S3)). The opposite signs of the transient signal are typically observed when hot-electron excitation causes a shift in the resonance. If the changes are primarily in the damping of the resonance, opposite signs are not expected. Which effect dominates depends on the Fresnel coefficients of the particular structure. In our case, the transient dynamics are influenced not only by hot-electron excitation in the nanorods and the mirror but also by the excitation of acoustic vibrations, which also alter the effective permittivity of the metamaterial. The transient reflection behaviour is therefore governed by the interplay between hot-electron-induced modifications of the permittivities of the nanorods and the mirror, and vibration-induced changes in the permittivities, all mediated through the Fresnel coefficients. At shorter wavelengths, a pronounced asymmetric Fano-type shape is present in the transient response, and this asymmetry manifests itself as an emergence of very fast (~300 fs) initial reflection changes observed in this spectral range, e.g., at wavelengths of 685 and 690 nm (Fig. 2b). The observed Fano shape in the time domain has strong spectral selectivity and sensitivity and results in substantial modifications of the relaxation time of the transient reflection across the spectral domain. A wavelength-independent oscillatory behaviour is also observed with the minima at approximately 7 and 19 ps post-excitation (after time-zero in Fig. 2b). Notably, no such temporal features are observed in the transient transmission of the metamaterial, and overall values of modulation amplitude in transmission remains lower despite the whole optical thickness of the metamaterial slab and the mirror playing a role in the response (Fig. 2c). The transient transmission decays monotonically in several picoseconds and is spectrally featureless. No oscillatory behaviour is observed in transmission, in stark contrast with the dynamics of the reflectance. This can be understood considering that the effect of the mirror–where the energy is absorbed under this excitation–is negligible in transmission (Fig. S2), so that heating of the mirror by the control pulse and associated excitation of acoustic vibrations affect reflection and have much less effect in transmission.

The observed spectral and wavelength dependences indicate the important role of the mirror in the process of redistribution of the energy within the system and the overall change of the reflection, producing the observed fast relaxation dynamics. The strong electron temperature increase in a mirror to which the metamaterial is connected may introduce a backward (from the mirror to the nanorods) hot-electron diffusion, which is usually not considered in the majority of the studies, despite similar geometries being are ubiquitous in nanofabrication. The pronounced rise of the electron temperature in the mirror drives a backward (mirror-to-nanorod) flux of hot electrons. The exact role of this process and the peculiarities of hot-carrier dynamics at the interface between the mirror and the nanorods depend on the values of interface thermal conductivity and electron diffusion between the mirror and the base of the nanorods, evaluation of which is challenging in real materials, but which is an important mechanism as demonstrated in this study and should not be overlooked. The thermal expansion of the mirror could also serve as the nanoscale transducer for the excitation of the rich spectrum of acoustic vibrations in the metamaterial, as will be discussed below.

**Fig. 3:**
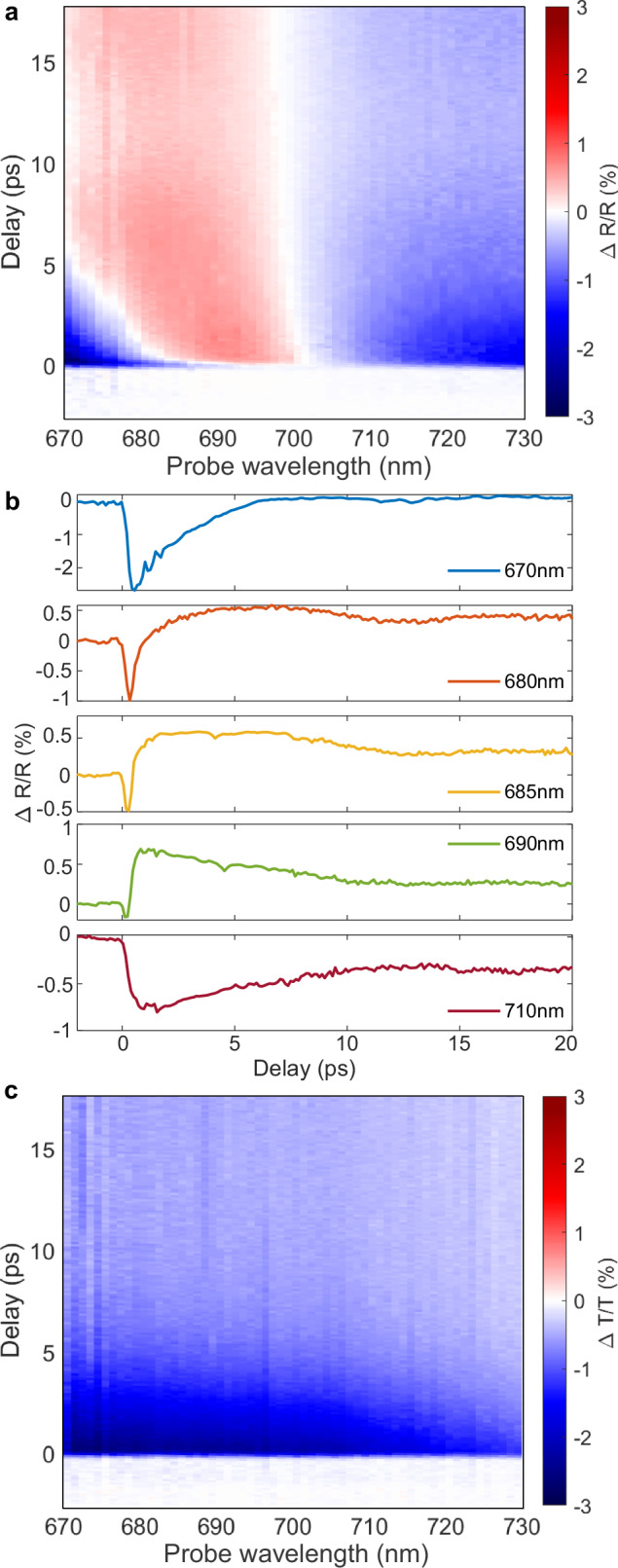
Transient optical spectra of the metamaterial under the 515 nm excitation. Transient (**a**, **b**) reflection and (**c**) transmission spectra measured with the excitation at a wavelength of 515 nm and a power of 10 mW, which is 5 times smaller than that of the IR pump in Fig. 2.

The situation is drastically different for a visible light excitation, which reduces electron heating in the mirror (Fig. 3). While the transient transmission mainly follows the same trend as observed with the NIR excitation, the reflection signal now undergoes a typical sign-changing behaviour, indicating the shift and broadening of a guided mode resonance of the metamaterial slab. The stronger electron heating, which in this case primarily takes place in the nanorods (Fig. 1e), results in the stronger modulation amplitude, compared to the NIR excitation: similar modulation amplitude is observed with 5 times smaller excitation intensity (Fig. 3). Overall, the transient signals beyond 20% in reflection have been observed with twice less power than under the NIR excitation. Interestingly, the electron temperature increase localised primarily in the nanorods also mitigates the optical damage to the sample, which was observed to be limited by the temperature increase in the gold mirror.

**Fig. 4:**
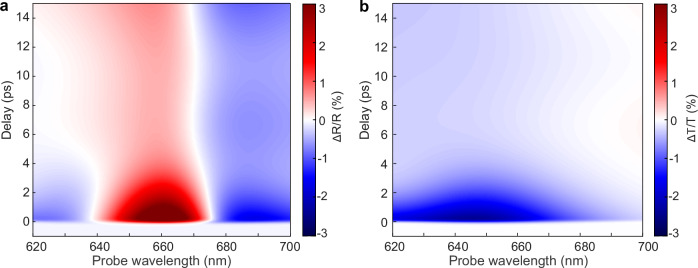
Numerical simulations of the transient response. Simulated transient (**a**) reflection and (**b**) transmission spectra of the metamaterial on a mirror system under the excitation at a wavelength of 515 nm.

The numerical modelling (see details in Methods) reproduces the overall transient behaviour in both transmission and reflection (Fig. 4a, b). We would like to emphasise that despite the success in modelling the response of a nanorod composite under visible illumination, the model faces challenges to replicate all features of the NIR-induced response, highlighting yet again the importance of detailed treatment of hot-carrier diffusion within optical metamaterials and between the nanorods and the mirror.

### Acoustic phonon properties

**Fig. 5:**
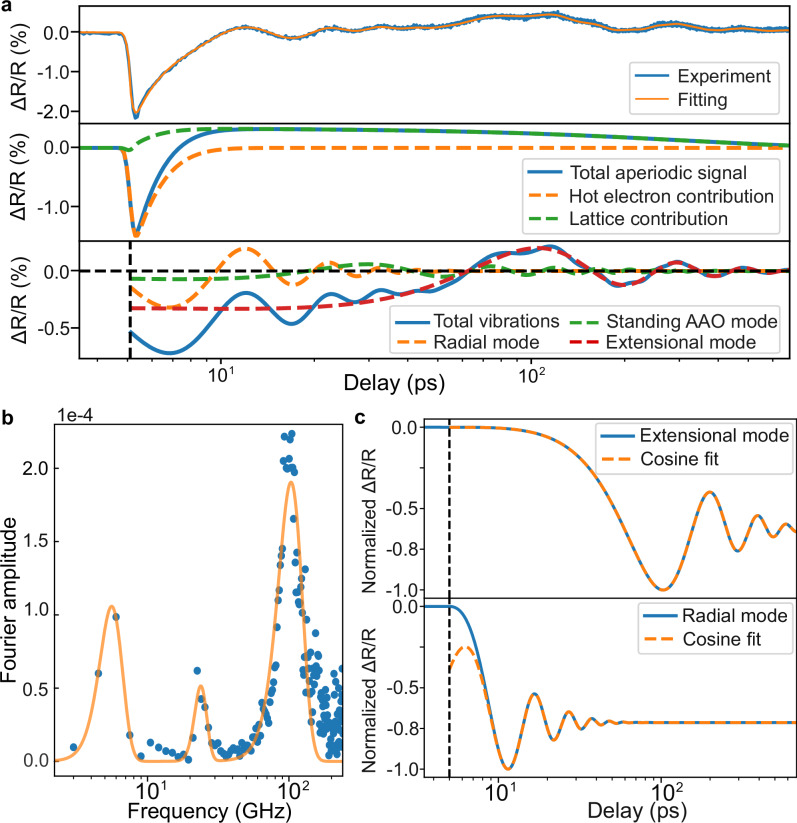
Spectrum of acoustic phonons. **a** Transient reflection trace at a wavelength of 680 nm under the 1030 nm excitation, with corresponding model fit (upper panel), separated into aperiodic (middle panel) and periodic (lower panel) parts. Log scale used for the horizontal axis for better display of features with different temporal scales. **b** Spectrum of the periodic part of (**a**) obtained with fast Fourier transform (orange line is Gaussian fit). **c** Solutions of Eq. (2) for (top) extensional mode of the nanorod and (bottom) radial breathing mode of the nanorod. Dashed orange lines represent cosine fits with different recovered initial phases.

The long-lived weakly wavelength-dependent oscillations in the transient response can be attributed to the excitation of various acoustic modes within the metamaterials (Fig. 2). Over the time scale of 500 ps, multiple superimposed periodic signals are observed featuring periods ranging from a few to hundreds of picoseconds (Fig. 5a). A detailed analysis of the frequencies of the modes done by the fast Fourier transform and least-square fitting (Fig. 5b) identified three damped oscillations with the frequencies of 5, 23 and 97 GHz and corresponding decay times of 190, 120 and 10 ps, respectively. We can identify the vibrations with the highest and lowest frequencies as fundamental breathing radial and extensional modes, respectively, of a weakly coupled individual nanorod in the metamaterial^26^:

1 $$\begin{array}{rcl}{\nu }_{br}&\,=&\,\frac{{\phi }_{0}c}{2\pi R},\\ {\nu }_{ext}&\,=&\,\frac{1}{2L}\sqrt{\frac{E}{\rho }},\end{array}$$

where *R* and *L* are the radius and length of the nanorod, *c*, *E*, and *ρ* are the longitudinal speed of sound, elastic modulus and density of gold, respectively, and the eigenvalue *ϕ*_0_ is the first root of the equation *ϕ*_*n*_*J*_0_(*ϕ*_*n*_) = (1 − 2*σ*)*J*_1_(*ϕ*_*n*_)/(1 − *σ*), with *σ* being the Poisson ratio, roughly equal to 2.28. Taking into consideration the characteristic dimensions of the metamaterial, this provides frequency estimates of 4.6 and 85 GHz, respectively, that match the experimental observations.

Since in the experiment the control and probe beams illuminate multiple nanorods, the decay times of these vibrations are mainly a signature of inhomogeneous broadening rather than intrinsic decay times which have been shown to be of the order of 100 and 400 ps for the breathing and extensional vibrations of isolated nanorods in air^26^ (the alumina matrix may also contribute to the broadening but on a smaller scale than the inhomogeneous broadening). The mode at the frequency of 23 GHz is consistent with the standing longitudinal acoustic wave in the alumina matrix (estimated frequency 22.7 GHz), with the period corresponding to the round trip across the film.

Two low frequency vibrational modes (extensional mode of the nanorods and standing wave in the matrix) are excited as a roughly cosine function with initial phases close to 20^∘^ as determined from the fit (Fig. 5a), consistent with previous observations^27–29^, signifying that the initial thermal expansion, that depends on the dynamics of electron and lattice temperatures happens on the timescale much faster than the period of the corresponding oscillation. The radial breathing mode of the nanorods, however, possesses an initial phase closer to 70^∘^. The latter could be understood by considering the simplistic dynamic model^29^:

2 $$\begin{array}{ll}\frac{{d}^{2}x}{d{t}^{2}}\,+\,\frac{2}{\tau }\frac{dx}{dt}+{\omega }_{0}^{2}x=\frac{A}{m}\sigma (t),\\ \sigma (t)\,=\,\gamma {C}_{l}\Delta {T}_{l}(t)-\frac{2}{3}\Delta {E}_{e}(t),\end{array}$$

where *x* is the vibration coordinate, *ω*_0_ and *τ* are the fundamental frequency and decay constant, respectively, that can be taken from fitting of experimental data, *A* and *m* are the area and mass of the nanorod, respectively, and *σ*(*t*) is the transient stress, created by the rise and decay of hot-electron and lattice temperatures. The latter contains two terms^30,31^: one caused by the lattice anharmonism proportional to the temperature of the lattice (*C*_*l*_ is the heat capacity of the lattice, *γ* ≈ −2.7 is the Grüneisen constant^31^), and another caused by the hot-electron pressure, proportional to the total energy *ΔE*_*e*_ stored in the hot-electron bath.

Solving Eq. (2) allows to determine the initial phases for extensional and radial vibrations of a gold nanorod, which are found to be around 12^∘^, 52^∘^, respectively, in a close agreement with the experimental observations (Fig. 5c). We do not apply this model for analysis of the standing acoustic wave in the composite, since it is not directly excited by the hot-electron dynamics, but rather through mechanical coupling with the other parts of the structure.

We would like to emphasise that such temporal shaping of transient response, observed under the NIR excitation, occurs only in reflection, where the optical resonance associated with the guided mode in the hyperbolic metamaterial composite is present, providing the necessary phase flip. In transmission, on the other hand, the optical modulation produces the changes in optical constants decaying much slower–on the order of a few picoseconds–consistent with the hot-electron decay in gold (Figs. 2c and 3b).

The difference in the spatial distributions of the energy absorption between the visible and NIR excitation regimes (Fig. 1d, e) plays an important role in determining the efficiency of the phonon excitation. Under the NIR excitation, a large portion of the absorbed energy is deposited in the mirror below the nanorods, which promotes more efficient excitation of the nanorod vibrational modes: the mirror acts as an acoustic membrane driving the nanorods. In contrast, for a visible excitation, when the pulse energy is predominantly absorbed in the nanorods, the phonon excitation is much weaker.

## Discussion

We studied a plasmonic nanorod metamaterial-on-a-mirror structure to demonstrate ultrafast all-optical processes and excitation of acoustic vibrational modes, mediated by the mirror and the leaky waveguided modes of the metamaterial slab. The ultrafast nonlinear optical response of plasmonic nanorod metamaterials was previously demonstrated in transmission only for TM-polarised light around the ENZ wavelength, where it is limited by strong absorption. The rich optical reflection spectrum of the metamaterial in the hyperbolic spectral range offers a variety of optical resonances, corresponding to both TE and TM guided modes intrinsic to the structure, which provides the polarisation diversity of the signal and control light. The differences in absorption magnitude and absorption spatial profiles along the nanorods at different wavelengths result in a strong sensitivity of the transient reflection to the plasmonic mirror effects and the acoustic vibrations.

By selecting the excitation wavelength, the energy distribution localised in the metamaterial or the substrate can be controlled, resulting in spectrally-tuneable, non-uniform hot-electron population and selective excitation of acoustic phonons. The strong, excitation-wavelength-dependent electron temperature increase in a mirror to which the metamaterial is connected is expected to introduce a backward hot-electron diffusion into the nanorods, important for both temporal characteristics of nonlinearity and influences the excitation of vibrational acoustic modes. Interestingly, the damage threshold is significantly reduced at the wavelengths for which absorption in a mirror dominates.

The nonlinear reflection exhibits the timescales shorter than the characteristic hot-carrier relaxation times in the metamaterial constituents. The fast reflection changes represent the wavelength-dependent behaviour, unlike the dynamics of optical transmission (cf. Fig. 2c left and right panels). The transient transmission, which is not influenced by acoustic vibrations, can be described by a single decaying exponent. The fast changes in reflection can be explained by the interplay of different transient contributions to optical reflectance, including hot-carrier mediated processes in the nanorods and the mirror, heat transfer between the mirror and the nanorods, lattice temperature, and acoustic phonon-modulated reflectance. The observed Fano-type asymmetric line shape originates from the interference of transient contributions to reflected light. To obtain the correct spectral shape, one has to assume a combination of complex transient reflectivities, some of which change phase by 2*π* around the resonance (as expected from the resonant metamaterial) and some being spectrally flat. These optical signals are the equivalent of localised and broad states in the Fano-type processes and represent a simple but unusual interference pattern in the spectral domain since static reflectivities do not manifest such behaviour. This makes it plausible that the spatial distribution of hot electrons along the nanorod length, the strong absorption in the mirror and the associated backward heat transfer, and acoustic modes are likely responsible for this effect, as they are different for NIR and visible photoexcitation (cf. Figs. 1d, e, 2 and 3). The interference of multiple time-dependent signals in this respect underpins the behaviour of the transient response of optically thick metamaterials acting as a distributed time-dependent reflector, different parts of which produce spectrally different contributions to the overall transient reflectivity that are allowed to interfere destructively in this case and form a Fano-type line shape.

The acoustic vibrations of the constituting elements of the metamaterial excited by the decay of hot carriers behave as a superposition of sine or cosine functions in time trajectory^29,32^. Such acoustic excitations have been extensively studied in both isolated nanoparticles in various environments^16,33^, where they could be used as nanoscale probes of local elasticity of materials and local viscosity of surrounding medium, in composite nanostructures where the design of the structure offers wavelength and polarisation sensitivity^34,35^, and as ultra-compact sources of surface acoustic waves.

The obtained results provide new insights into the understanding of light–matter interactions in complex nanostructured media and expand the potential for designing nonlinear optical properties for nanophotonic and acousto-optic applications, time-varying optics, and nanoscale metrology with acoustic vibrations.

## Methods

### Sample fabrication

**Fig. 6:**
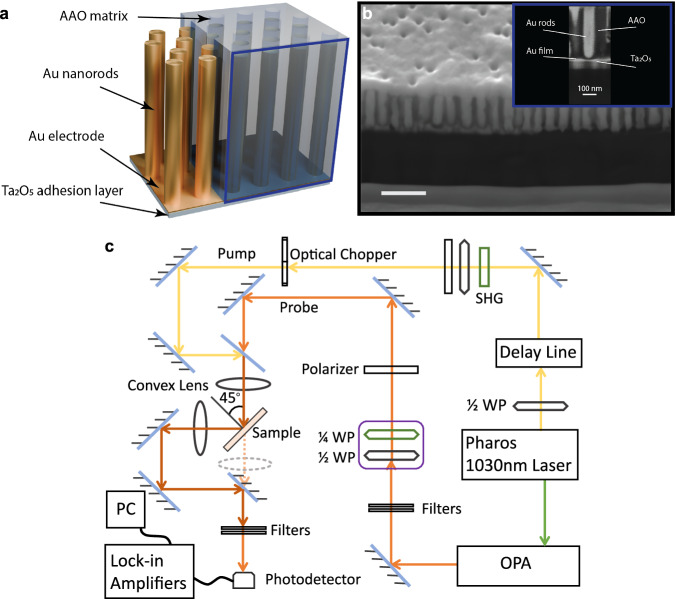
The metamaterial structure and the experimental setup. **a** Schematic and **b** the SEM image of the cross-sectional view of the metamaterial (scale bar is 200 nm); insert is the cross-section image of a single nanorod. **c** Schematic diagram of the pump-probe spectroscopy setup (green box indicates a removable SHG module for generation of a 515 nm light).

The plasmonic anisotropic metamaterial based on an array of gold nanorods (Fig. 6a, b) was fabricated using the alumina template method^18,36^. Thin aluminium films of up to 400 nm thickness were first anodised in 0.3 M sulphuric acid at 25 V to create a porous anodised aluminium oxide (AAO) template. After removing the barrier layer with a 30 mM NaOH etching solution, gold nanorods were electrochemically grown within a substrate-supported alumina template, which included a sputtered 10 nm Ta_2_O_5_ adhesion layer and an 8 nm Au film acting as the working electrode for electrochemical deposition. The diameter and periodicity of the Au nanorods were determined by the geometry of the AAO templates and were controlled by anodisation conditions such as the choice of electrolyte, its temperature, and the anodisation voltage. The length of the nanorod structures was controlled by the duration of the electrochemical deposition.

### Time-resolved measurements

The time-resolved transient measurements were performed using an optical parametric amplifier (OPA) to provide a tuneable probe beam with the pulse duration of 150 fs. The OPA is pumped by an Yb- amplified laser system (Light Conversion Pharos), which also provided a pump beam at a wavelength of 1030 nm and ≈250 fs pulse duration (Fig. 6c). The output of the pump laser was frequency doubled in an additional Barium Borate crystal to realise the excitation at the wavelength of 515 nm. The TM-polarised excitation pulses and TE-polarised probe pulses were incident on the sample at an angle of 45°. The experiment is designed to allow measurements of both transient reflection and transmission.

### Modelling electron dynamics

To model the electron and lattice dynamics in the metamaterial, we employed the two temperature model (TTM) to describe the equilibrium hot-electron population, established by initial thermalisation through ultrafast electron-electron collisions, that subsequently transfers energy to the crystal lattice and the environment through electron-phonon and phonon-phonon scattering^28,37^. TTM is applicable for modelling the hot-electron dynamics in gold at both excitation wavelengths, as non-parabolicity of electron bands does not play a significant role^5^.

Due to the lateral cross-section of an individual nanorod (≈28 nm) being much smaller than the sizes of the pump and probe beams and a skin-depth in gold at both the pump and probe wavelengths, we ignore the in-plane variation of electron and lattice temperatures. This leads to the one-dimensional model, that without the loss of generality, only needs to consider the heat transfer between the thermodynamic ensembles and 1D heat diffusion:

3 $${C}_{e}({T}_{e})\frac{\partial {T}_{e}}{\partial t}=\nabla ({k}_{e}\nabla {T}_{e})-G({T}_{e}-{T}_{l})+S(t,z),$$

4 $${C}_{l}\frac{\partial {T}_{l}}{\partial t}=G({T}_{e}-{T}_{l}),$$

where *C*_*e*_ and *C*_*l*_ are the heat capacities of the electron and lattice subsystems, respectively, *k*_*e*_, *G* and *S*(*t*, *z*) are the electron thermal conductivity, the electron–phonon coupling factor, and the source term describing electron heating by the laser pulse, as a function of the time^38^.

At hot-electron temperatures much smaller than the Fermi temperature, *C*_*e*_ can be approximated by a linear function *C*_*e*_ = *C*_*e*0_*T*_*e*_, with the proportionality constant *C*_*e*0_ = *π*^2^*Nk*_*B*_/2*T*_*F*_, following from the Sommerfeld theory of metals, where *N* is the number density of atoms, *k*_*B*_ is the Boltzmann constant, *T*_*F*_ is the Fermi temperature^39^. The heat capacity of the lattice in taken in the Dulong-Petit limit, considering the low Debye temperature of gold (around 170 K). For gold, *C*_*e*0_ = 70 J m^−3 ^K^−2^, *C*_*l*_ = 2.5 × 10^6^ J m^−3 ^K^−1^, *G* = 2.6 × 10^16^ W m^−3 ^K^−1^, and *T*_*F*_ = 6.42 × 10^4^ K^38–40^.

The source term, appearing in Eq. (3), can be consequently described by the following expression:

5 $$S(t,z)=\sqrt{\frac{4\ln 2}{\pi }}\frac{\alpha (z)}{{t}_{p}}\phi \cdot {e}^{-4\ln 2{(t/{t}_{p})}^{2}},$$

where *ϕ* is the laser fluence, *α*(*z*) is the spectrally integrated absorption coefficient as a function of depth in the material, and *t*_*p*_ is the duration of a laser pulse taken at half maximum^41^.

We have rigorously solved the set of coupled Eqs. (3-5) in the finite-volume PDE solver available in the FiPy package^42^, and compared the results with the solution of ‘local’ TTM, that does not include the diffusion term:

6 $${C}_{e0}{T}_{e}\frac{d{T}_{e}}{dt}=G({T}_{l}-{T}_{e})+S(t),$$

7 $${C}_{l}\frac{d{T}_{l}}{dt}=G({T}_{e}-{T}_{l}).$$

The latter approach offers substantial ease of the computational complexity at the expense of ignoring the heat diffusion along the nanorods in the composite. While previous results have outlined the importance of accounting for the diffusion in some cases^9^, our results suggest that, for the excitation wavelength used in the modelling for this work (515 nm), the full solution is excessive and does not offer additional benefits. Due to this finding, we employed the model described by Eq. (6), solved independently for the nanorods and gold underlayer, to generate the transient spectra presented in this work.

### Modelling transient optical response

To model the transient permittivity of gold, we employed a two-band model^43^ and a procedure described in ref. ^44^. Within this approach, the permittivity of gold *ϵ*_*Au*_ = *ϵ*_*inter*_ + *ϵ*_*intra*_ is separated into the single particle interband contribution, explicitly taking into account the optical transitions around X and L points of the band structure of gold, and a free-electron contribution, given by the Drude model:

8 $$\Im [{\epsilon }_{inter}(\hslash \omega ,t)]=\frac{{A}_{X}{J}_{X}(\hslash \omega ,t)+{A}_{{L}_{4}^{+}}{J}_{{L}_{4}^{+}}(\hslash \omega ,t)+{A}_{{L}_{5+6}^{+}}{J}_{{L}_{5+6}^{+}}(\hslash \omega ,t)}{{(\hslash \omega )}^{2}},$$

9 $${\epsilon }_{intra}={\epsilon }_{\inf }-\frac{{\omega }_{p}^{2}}{{\omega }^{2}+i\omega \Gamma (\hslash \omega ,{T}_{e},{T}_{l})},$$

where *ω*_*p*_ is the bulk plasma frequency, *A*_*i*_ are the spectral weights of different interband transitions, and *J*_*i*_ are the joint densities of states calculated by integrating the energy distribution of the joint density of states (EDJDOS) with the Fermi-Dirac occupation factors at a given electron temperature^43,45^. The damping *Γ*(*ℏω*, *T*_*e*_, *T*_*l*_) = *Γ*_*e*−*e*_(*ℏω*, *T*_*e*_) + *Γ*_*e*−*ph*_(*T*_*l*_) contains the terms associated with the fractional Umklapp electron–electron and electron–phonon scatterings given by, respectively:

10 $${\Gamma }_{e-e}(\hslash \omega ,{T}_{e})=\frac{{\pi }^{3}\beta \Delta }{12\hslash {E}_{F}}\left[{({k}_{B}{T}_{e})}^{2}+{(\hslash \omega /2\pi )}^{2}\right],$$

11 $${\Gamma }_{e-ph}({T}_{l})=\frac{1}{{\tau }_{0}}\left[\frac{2}{5}+4{\left(\frac{{T}_{l}}{\Theta }\right)}^{5}\mathop{\int}\nolimits_{0}^{\Theta /{T}_{l}}\frac{{z}^{4}}{{e}^{z}-1}dz\right],$$

where *Δ* = 0.75 and *β* = 0.55 are dimensionless constants, characterising the electron–electron scattering, typical for noble metals^46^, *θ* = 170 K is the Debye temperature, and *E*_*F*_ = 5.53 eV is the Fermi energy.

The real part of the interband dielectric function of gold was obtained using the Kramers–Kronig transformation, and parameters *A*_*i*_, *ϵ*_*inf*_ and *τ*_0_ were varied to match the tabulated values for the permittivity of gold at room temperature^47^.

Additionally, in order to represent the electrodeposited gold of the nanorods, that is known to demonstrate higher absorption than pure gold films due to polycrystallinity, we incorporated the ‘restricted’ mean free path *R* for the free electrons into the dynamic model by augmenting the microscopic dielectric function of gold^47^:

12 $${\epsilon }_{Au}^{R}={\epsilon }_{Au}+\frac{i{\omega }_{p}^{2}\Gamma (L-R)}{\omega (\omega +i\Gamma )(\omega R+i\Gamma L)},$$

where *L* = 35 nm is the mean free path, corresponding to bulk gold. In accordance with the previous results on similar experimental samples, we set *R* = 13 nm for the studied samples.

Transfer matrix model was used to simulate the optical response of the metamaterial in the local effective medium approximation^18^, which was checked to be valid for the metamaterial with the studied parameters, for which the effects of spatial dispersion are negligible.

In order to qualitatively incorporate the effect of phonon vibrations in the simulated transient spectra, we used the numerical solutions of Eq. (2), parameterised with the experimental values for normal frequencies and acoustic damping. The breathing and extensional vibrations were assumed to contribute to the optical response by slightly modifying the filling factor and the rod length, respectively. Therefore, an additional term in the transient response was added according to

13 $$\frac{\Delta F}{F}=A\frac{\partial F(\lambda ,r,l)}{\partial r}\Delta r(t)+B\frac{\partial F(\lambda ,r,l)}{\partial l}\Delta l(t),$$

where *F* is either the reflection or transmission coefficient of the metamaterial, *Δr*(*t*) and *Δl*(*t*) are the time-dependent radius and length of nanorods, respectively, and A and B are fitting constants.

## Supplementary information

Supplementary Information

## Data Availability

All the data supporting the findings of this work are presented in the results section and available from the corresponding author upon request.
