# Supplementary material for: Spectral synthesis of temporal response of nonlinearity through tuneable electron and phonon dynamics in a metamaterial
Source: Npj Nanophoton. 2026 Jan 8;3(1):2. doi: 10.1038/s44310-025-00098-x (PMC12783046; doi:10.1038/s44310-025-00098-x)
Supplement: Supplementary file 1 — Supplementary Information [file 44310_2025_98_MOESM1_ESM.pdf]

Supplementary Information

Spectral synthesis of temporal response of nonlinearity through tuneable electron and phonon dynamics in a metamaterial

Jingyi Wu[1*], Anton Yu. Bykov[1], Anastasiia Zaleska[1], Anatoly V. Zayats[1]

[1]Department of Physics and London Centre for Nanotechnology, King's College London, Strand, London, WC2R 2LS, UK.

*Corresponding author(s). E-mail(s): jingyi.2.wu@kcl.ac.uk;

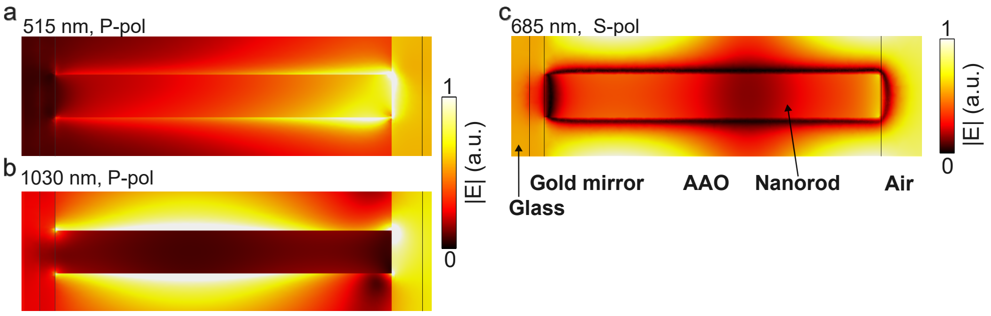

Fig. S1 Spatial distributions of the electric field norm in a unit cell of the metamaterial in a ground state at (a) 515 nm and (b) 1030 nm excitation wavelength for a p-polarised pump light and (c) 685 nm wavelength for s-polarised probe light. The fields are normalised independently in each panel to allow for direct comparison. The angle of incidence is 45°. The metamaterial parameters are as in Fig. 1.

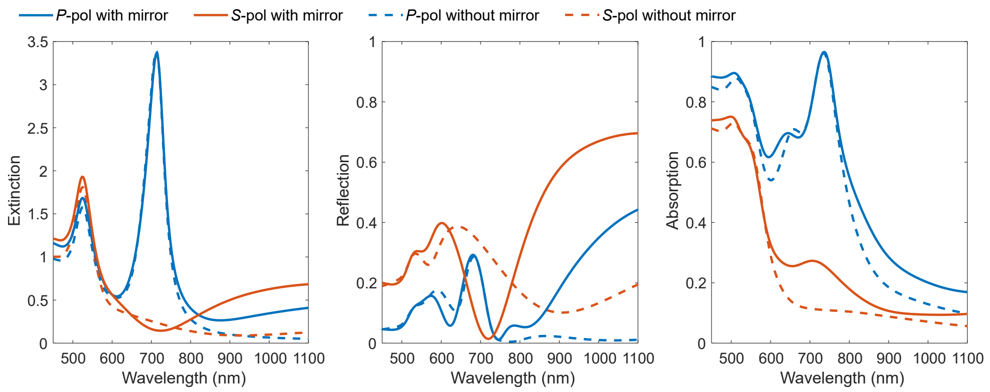

Fig. S2 Extinction, reflection and absorption spectra of the nanorod metamaterial on a gold mirror and in the absence of a mirror for both p- and s-polarisations of light, simulated using the effective medium theory. The angle of incidence is 45°.

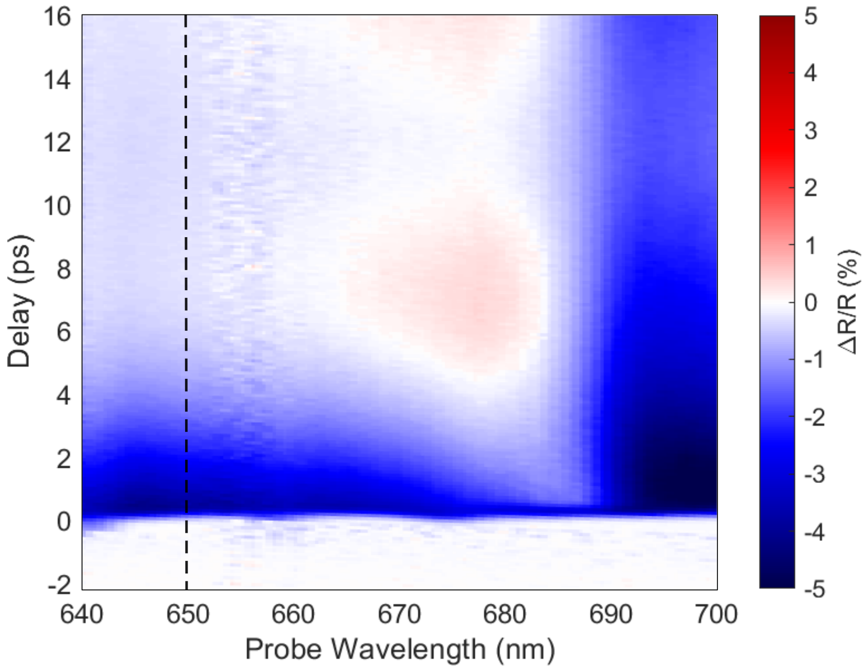

Fig. S3 Transient reflection spectra measured with the 1030 nm excitation wavelength. The ENZ wavelength of the metamaterial is indicated by a dashed line. Stronger modulation is observed away from the ENZ wavelength at the reflection resonance.
